# Crystal structure of 4,6-di­amino-2,2-dimethyl-3-[3-(2,4,5-tri­chloro­phen­oxy)prop­oxy]-2,3-di­hydro-1,3,5-triazin-1-ium chloride methanol monosolvate

**DOI:** 10.1107/S205698901501378X

**Published:** 2015-07-29

**Authors:** Pattarapol Khongsuk, Samran Prabpai, Palangpon Kongsaeree

**Affiliations:** a Department of Chemistry and Center of Excellence for Innovation in Chemistry, and Center for Excellence in Protein Structure and Function, Faculty of Science, Mahidol University, Bangkok 10400, Thailand

**Keywords:** crystal structure, triazine, anti­folate drug, anti­malarial, hydrogen bonding

## Abstract

In the title methanol-solvated salt, C_14_H_19_Cl_3_N_5_O_2_^+^·Cl^−^·CH_3_OH, the triazine mol­ecule is protonated at one of the triazine N atoms. In the crystal, the triazine cations are linked through a pair of N—H⋯N hydrogen bonds, with graph-set *R*_2_^2^(8), forming an inversion dimer. The protonated N atom and the 2- and 4-amino groups of the triazine cation inter­act with the chloride anion through N—H⋯Cl hydrogen bonds, leading to the formation of a tape structure running along the *b-*axis direction. A short Cl⋯Cl contact [3.2937 (9) Å] is observed in the tape. The methanol mol­ecule is linked to the chloride anion and the triazine cation, respectively, by an O—H⋯Cl hydrogen bond and a C—H⋯O inter­action.

## Related literature   

For anti­folate anti­malarial drugs, see: Toyoda *et al.* (1997 ▸); Yuthavong (2002 ▸). For anti­folate drug resistance, see: Nzila (2006 ▸); Rieckmann *et al.* (1996 ▸). For our previous work on the protein crystallographic analysis of di­hydro­folate reductase, see: Yuvaniyama *et al.* (2003 ▸); Kongsaeree *et al.* (2005 ▸).
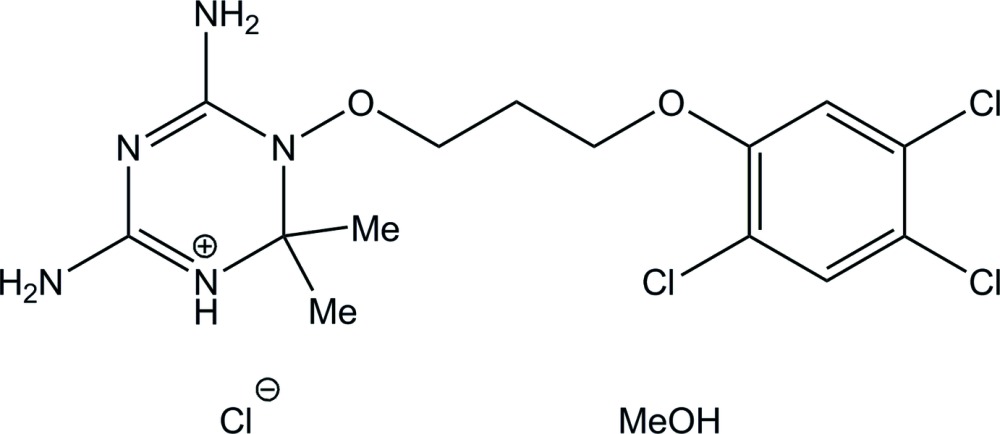


## Experimental   

### Crystal data  

C_14_H_19_Cl_3_N_5_O_2_^+^·Cl^−^·CH_4_O
*M* *_r_* = 463.18
Triclinic, 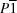

*a* = 8.5930 (4) Å
*b* = 9.3510 (3) Å
*c* = 14.6970 (7) Å
α = 75.422 (3)°
β = 78.2260 (19)°
γ = 70.194 (3)°
*V* = 1066.13 (8) Å^3^
*Z* = 2
Mo *K*α radiation
μ = 0.58 mm^−1^
*T* = 298 K
0.32 × 0.20 × 0.18 mm

### Data collection  

Nonius KappaCCD diffractometer
5032 measured reflections
2935 independent reflections
2681 reflections with *I* > 2σ(*I*)
*R*_int_ = 0.017
θ_max_ = 23.3°

### Refinement  

*R*[*F* ^2^ > 2σ(*F* ^2^)] = 0.034
*wR*(*F* ^2^) = 0.087
*S* = 1.06
2935 reflections
268 parameters
6 restraints
H atoms treated by a mixture of independent and constrained refinement
Δρ_max_ = 0.26 e Å^−3^
Δρ_min_ = −0.26 e Å^−3^

### 

Data collection: *KappaCCD Software* (Nonius, 1999 ▸); cell refinement: *SCALEPACK* (Otwinowski & Minor, 1997 ▸); data reduction: *DENZO* (Otwinowski & Minor, 1997 ▸); program(s) used to solve structure: *SIR97* (Altomare *et al.*, 1999 ▸); program(s) used to refine structure: *SHELXL2013* (Sheldrick, 2015 ▸); molecular graphics: *PLATON* (Spek, 2009 ▸); software used to prepare material for publication: *publCIF* (Westrip, 2010 ▸).

## Supplementary Material

Crystal structure: contains datablock(s) I, New_Global_Publ_Block. DOI: 10.1107/S205698901501378X/is5407sup1.cif

Structure factors: contains datablock(s) I. DOI: 10.1107/S205698901501378X/is5407Isup2.hkl

Supporting information file. DOI: 10.1107/S205698901501378X/is5407Isup3.cml

**Fig. 1.**
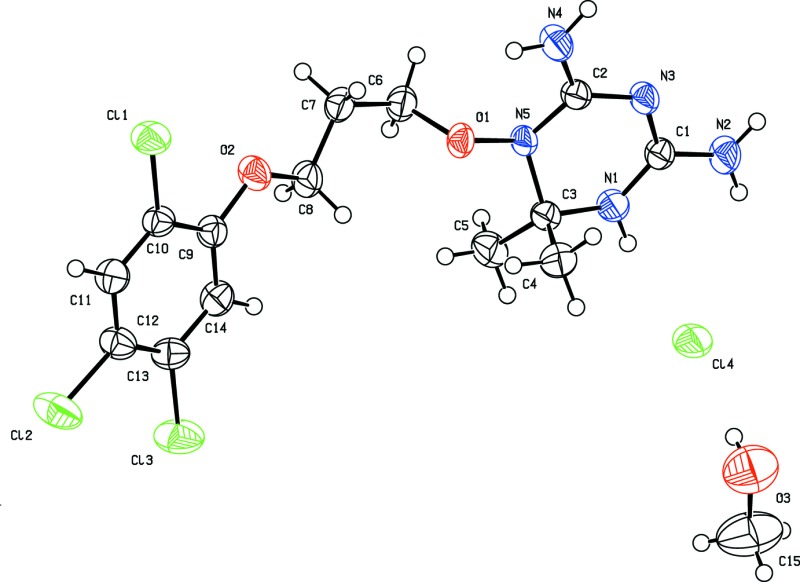
View of the molecular structure of the title compound, with displacement ellipsoids drawn at the 50% probability level.

. DOI: 10.1107/S205698901501378X/is5407fig1.tif
View of the mol­ecular structure of the title compound, with displacement ellipsoids drawn at the 50% probability level.

**Fig. 2.**
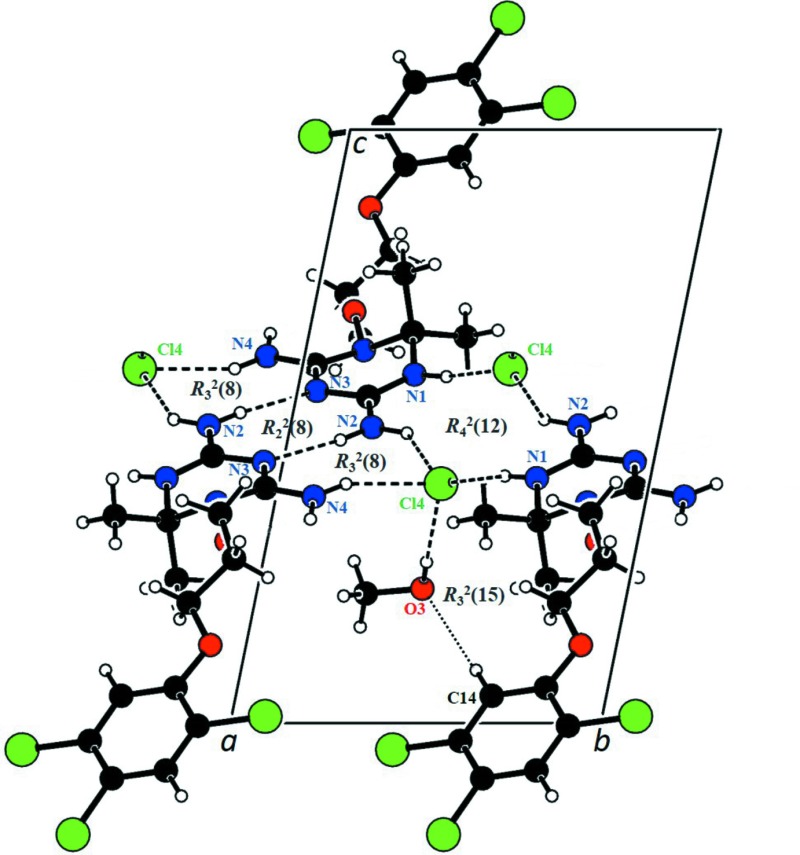
Hydrogen bonding interactions, showing molecules linked through N—H···N, N—H···Cl and O—H···Cl (dashed lines), and C—H···O (dotted line) hydrogen bonds.

. DOI: 10.1107/S205698901501378X/is5407fig2.tif
Hydrogen bonding inter­actions, showing mol­ecules linked through N—H⋯N, N—H⋯Cl and O—H⋯Cl (dashed lines), and C—H⋯O (dotted line) hydrogen bonds.

CCDC reference: 1414045

Additional supporting information:  crystallographic information; 3D view; checkCIF report

## Figures and Tables

**Table 1:** Hydrogen-bond geometry (, )

*D*H*A*	*D*H	H*A*	*D* *A*	*D*H*A*
N1H1Cl4^i^	0.87(1)	2.27(1)	3.1236(17)	167(2)
N2H2*A*Cl4^ii^	0.87(1)	2.64(2)	3.3285(17)	137(2)
N2H2*B*N3^iii^	0.87(1)	2.26(1)	3.122(2)	170(2)
N4H4*A*Cl4^iv^	0.88(1)	2.31(1)	3.1419(19)	158(2)
O3H3Cl4	0.82	2.35	3.166(2)	176
C14H14O3^v^	0.93	2.53	3.423(3)	161

Symmetry codes: (i) 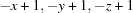; (ii) 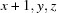; (iii) 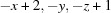; (iv) 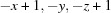; (v) 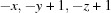.

## supplementary crystallographic information

### S1. Comment

The structure of the methanol-solvated salt compound, C_14_H_19_Cl_3_N_5_O_2_^+^.Cl^-^.CH_3_OH, was determined as part of a structural study of our dihydrofolate reductase (DHFR) in complex with its antifolate inhibitors. WR99210 {systematic name: 6,6-dimethyl-1-[3-(2,4,5-trichlorophenoxy)propoxy]-1,6-dihydro-1,3,5-triazine-2,4-diamine} is a potent inhibitor of dihydrofolate reductase enzyme (DHFR). With a structural resemblance with cycloguanil (Cyc), a metabolite of the antimalarial drug proguanil (Toyoda *et al.*, 1997), WR99210 has a flexible propoxy linker and a phenyl group with three chlorine atoms (Yuthavong, 2002; Nzila, 2006). Studies in animal models demonstrated a low bioavailability of WR99210, preventing its further development as an antimalarial agent (Rieckmann *et al.*, 1996). The crystal structures of the bifunctional *Plasmodium falciparum* DHFR-TS and the monofunctional *P. vivax* DHFR have been reported in complex with WR99210, and with pyrimethamines, respectively (Yuvaniyama *et al.*, 2003; Kongsaeree *et al.*, 2005). The protein structures complexed with WR99210 have provided valuable insight into interdomain interactions and also opened a new dimension in the design of new drugs to fight against malaria. Herein, we report a single crystal X-ray structure of 4,6-diamino-2,2-dimethyl-3-[3-(2,4,5-trichlorophenoxy)propoxy]-2,3-dihydro-1,3,5-triazin-1-ium chloride methanol solvate, (I).

In the title compound, the WR99210 molecule is protonated at one of the nitrogen atoms of the triazine moiety. This is evident from the increase in the internal angle at protonated N1 [C1—N1—C3 = 122.30 (16) Å] compared with that of the unprotanated atoms N3 [C2—N3—C1 = 116.03 (16) Å] and N5 [C2—N5—C3 = 117.77 (15) Å]. The triazine ring adopts the conformation described as an intermediate between a flatten screw-boat and a half-chair with C3 atom. The angle between the geminal flagpole and bowsprit methyl groups is 112.31 (18)°. In addition, the propoxy linker between the triazine and the trichlorophenyl group allowed free rotations of *sp*^3^-hybridized C6, C7 and C8 atoms (Fig. 1).

In the crystal, the triazine moiety is centrosymmetrically paired through N—H···N hydrogen bonds involving the 2-amino group and the N3 atom of the triazine, leading to a hydrogen-bonding pattern with a graph-set *R*_2_^2^(8) (Fig. 2). The pairs further interact with the chloride anion through N—H···Cl hydrogen bonds. The chloride anion connects 2-amino and 4-amino groups on either side of the paired triazine, forming an eight-membered hydrogen bonded ring motif with a graph-set *R*_3_^2^(8). The protonated N1 and 2-amino groups of the cationic triazine also interacted with two chloride anions to form a ring motif with a graph-set *R*_4_^2^(12). In addition, we found a graph-set *R*_3_^2^(15) ring motif through an O—H···Cl hydrogen bond between the methanol molecule and the chloride anion as well as a C—H···O hydrogen bond between the benzene ring (C14) and the methanol molecule (Table 1).

### S2. Experimental

WR99210 was a kind gift from Dr. Bongkoch Tarnchompoo, BIOTEC, National Science and Technology Development Agency, Thailand. Single crystals of the title compound were prepared from a methanolic solution by slow evaporation at 298 K. The colorless crystals suitable for X-ray diffraction were obtained after a few days.

### S3. Refinement

The N-bound H atoms were located in a difference Fourier map and were refined with restraint of N—H = 0.88 (1) Å. All other H atoms were placed in idealized positions and refined as riding atoms, with C—H = 0.93–0.97 Å and O—H = 0.82 Å, and with *U*_iso_(H) = 1.5*U*_eq_(O, C_methyl_) and 1.2*U*_eq_(C) for other H atoms.

### Crystal data

**Table:**

C_14_H_19_Cl_3_N_5_O_2_^+^·Cl^−^·CH_4_O	*Z* = 2
*M**_r_* = 463.18	*F*(000) = 480
Triclinic, *P*1	*D*_x_ = 1.443 Mg m^−^^3^
*a* = 8.5930 (4) Å	Mo *K*α radiation, λ = 0.71073 Å
*b* = 9.3510 (3) Å	Cell parameters from 2724 reflections
*c* = 14.6970 (7) Å	θ = 2.9–23.3°
α = 75.422 (3)°	µ = 0.58 mm^−^^1^
β = 78.2260 (19)°	*T* = 298 K
γ = 70.194 (3)°	Rod, colourless
*V* = 1066.13 (8) Å^3^	0.32 × 0.20 × 0.18 mm

### Data collection

**Table:**

Nonius KappaCCD diffractometer	2681 reflections with *I* > 2σ(*I*)
Radiation source: fine-focus sealed tube	*R*_int_ = 0.017
Detector resolution: 9 pixels mm^-1^	θ_max_ = 23.3°, θ_min_ = 3.1°
CCD scans	*h* = −9→9
5032 measured reflections	*k* = −10→9
2935 independent reflections	*l* = −16→16

### Refinement

**Table:**

Refinement on *F*^2^	Primary atom site location: structure-invariant direct methods
Least-squares matrix: full	Secondary atom site location: difference Fourier map
*R*[*F*^2^ > 2σ(*F*^2^)] = 0.034	Hydrogen site location: mixed
*wR*(*F*^2^) = 0.087	H atoms treated by a mixture of independent and constrained refinement
*S* = 1.06	*w* = 1/[σ^2^(*F*_o_^2^) + (0.0351*P*)^2^ + 0.5001*P*] where *P* = (*F*_o_^2^ + 2*F*_c_^2^)/3
2935 reflections	(Δ/σ)_max_ = 0.001
268 parameters	Δρ_max_ = 0.26 e Å^−^^3^
6 restraints	Δρ_min_ = −0.26 e Å^−^^3^

### Special details

**Table:**

Geometry. All e.s.d.'s (except the e.s.d. in the dihedral angle between two l.s. planes) are estimated using the full covariance matrix. The cell e.s.d.'s are taken into account individually in the estimation of e.s.d.'s in distances, angles and torsion angles; correlations between e.s.d.'s in cell parameters are only used when they are defined by crystal symmetry. An approximate (isotropic) treatment of cell e.s.d.'s is used for estimating e.s.d.'s involving l.s. planes.
Refinement. Refinement of *F*^2^ against ALL reflections. The weighted *R*-factor *wR* and goodness of fit *S* are based on *F*^2^, conventional *R*-factors *R* are based on *F*, with *F* set to zero for negative *F*^2^. The threshold expression of *F*^2^ > *σ*(*F*^2^) is used only for calculating *R*-factors(gt) *etc*. and is not relevant to the choice of reflections for refinement. *R*-factors based on *F*^2^ are statistically about twice as large as those based on *F*, and *R*-factors based on ALL data will be even larger.

### Fractional atomic coordinates and isotropic or equivalent isotropic displacement parameters (Å^2^)

**Table:**

	*x*	*y*	*z*	*U*_iso_*/*U*_eq_	
Cl1	−0.26674 (7)	−0.09037 (6)	0.98859 (4)	0.05048 (18)	
Cl2	−0.50317 (9)	0.36361 (7)	1.18779 (4)	0.0669 (2)	
Cl3	−0.27290 (9)	0.54749 (7)	1.04479 (5)	0.0658 (2)	
Cl4	0.17598 (7)	0.43814 (6)	0.40327 (4)	0.05568 (19)	
O1	0.36692 (15)	0.10928 (15)	0.69350 (9)	0.0364 (3)	
O2	−0.07327 (17)	0.09663 (16)	0.86849 (10)	0.0423 (3)	
O3	0.0138 (3)	0.4448 (3)	0.22657 (17)	0.0928 (7)	
H3	0.0601	0.4417	0.2709	0.139*	
N1	0.6726 (2)	0.30658 (19)	0.58733 (12)	0.0404 (4)	
N2	0.9243 (2)	0.2208 (2)	0.49858 (14)	0.0459 (4)	
N3	0.77155 (19)	0.05192 (18)	0.56004 (11)	0.0369 (4)	
N4	0.5995 (2)	−0.1015 (2)	0.61841 (14)	0.0491 (5)	
N5	0.49520 (18)	0.15639 (17)	0.62848 (11)	0.0327 (4)	
C1	0.7877 (2)	0.1939 (2)	0.54981 (13)	0.0338 (4)	
C2	0.6256 (2)	0.0339 (2)	0.60370 (13)	0.0334 (4)	
C3	0.5346 (2)	0.2785 (2)	0.65866 (14)	0.0356 (4)	
C4	0.5862 (3)	0.2234 (3)	0.75760 (15)	0.0501 (5)	
H4C	0.4938	0.2043	0.8026	0.060*	
H4D	0.6195	0.3015	0.7732	0.060*	
H4E	0.6779	0.1294	0.7596	0.060*	
C5	0.3872 (3)	0.4247 (2)	0.65107 (18)	0.0509 (6)	
H5A	0.2982	0.4096	0.7001	0.061*	
H5B	0.3498	0.4478	0.5903	0.061*	
H5C	0.4200	0.5094	0.6580	0.061*	
C6	0.2207 (2)	0.1419 (3)	0.64724 (14)	0.0422 (5)	
H6A	0.2490	0.0902	0.5939	0.051*	
H6B	0.1740	0.2525	0.6248	0.051*	
C7	0.0993 (2)	0.0797 (3)	0.72291 (15)	0.0421 (5)	
H7A	0.0103	0.0749	0.6935	0.051*	
H7B	0.1564	−0.0249	0.7532	0.051*	
C8	0.0247 (3)	0.1781 (2)	0.79714 (14)	0.0418 (5)	
H8A	0.1119	0.1912	0.8241	0.050*	
H8B	−0.0448	0.2796	0.7698	0.050*	
C9	−0.1674 (2)	0.1672 (2)	0.94055 (13)	0.0348 (4)	
C10	−0.2683 (2)	0.0871 (2)	1.00452 (13)	0.0349 (4)	
C11	−0.3698 (3)	0.1481 (2)	1.07983 (14)	0.0405 (5)	
H11	−0.4368	0.0939	1.1217	0.049*	
C12	−0.3719 (3)	0.2895 (2)	1.09306 (14)	0.0419 (5)	
C13	−0.2726 (3)	0.3696 (2)	1.02998 (14)	0.0418 (5)	
C14	−0.1711 (3)	0.3094 (2)	0.95396 (14)	0.0407 (5)	
H14	−0.1053	0.3647	0.9118	0.049*	
C15	0.0334 (5)	0.2948 (4)	0.2183 (3)	0.1067 (12)	
H15A	−0.0107	0.2981	0.1625	0.160*	
H15B	−0.0254	0.2456	0.2732	0.160*	
H15C	0.1498	0.2369	0.2133	0.160*	
H1	0.700 (3)	0.3881 (18)	0.5866 (17)	0.053 (7)*	
H2A	0.944 (3)	0.3083 (16)	0.4926 (16)	0.045 (6)*	
H2B	1.002 (2)	0.144 (2)	0.4770 (16)	0.052 (7)*	
H4A	0.680 (2)	−0.181 (2)	0.6007 (17)	0.057 (7)*	
H4B	0.507 (2)	−0.108 (3)	0.6546 (16)	0.067 (8)*	

### Atomic displacement parameters (Å^2^)

**Table:**

	*U*^11^	*U*^22^	*U*^33^	*U*^12^	*U*^13^	*U*^23^
Cl1	0.0612 (4)	0.0386 (3)	0.0568 (3)	−0.0254 (3)	0.0059 (3)	−0.0153 (2)
Cl2	0.0924 (5)	0.0554 (4)	0.0519 (4)	−0.0290 (3)	0.0215 (3)	−0.0243 (3)
Cl3	0.0963 (5)	0.0422 (3)	0.0678 (4)	−0.0344 (3)	0.0018 (3)	−0.0180 (3)
Cl4	0.0613 (4)	0.0410 (3)	0.0719 (4)	−0.0260 (3)	0.0097 (3)	−0.0241 (3)
O1	0.0292 (7)	0.0467 (8)	0.0340 (7)	−0.0173 (6)	0.0032 (5)	−0.0070 (6)
O2	0.0422 (8)	0.0407 (8)	0.0426 (8)	−0.0178 (6)	0.0095 (6)	−0.0107 (6)
O3	0.1093 (18)	0.0790 (15)	0.0964 (17)	−0.0336 (13)	−0.0224 (13)	−0.0144 (12)
N1	0.0407 (9)	0.0317 (9)	0.0515 (10)	−0.0178 (8)	0.0047 (8)	−0.0123 (8)
N2	0.0406 (10)	0.0400 (11)	0.0577 (11)	−0.0220 (9)	0.0091 (8)	−0.0093 (9)
N3	0.0341 (9)	0.0332 (9)	0.0432 (9)	−0.0151 (7)	0.0078 (7)	−0.0109 (7)
N4	0.0443 (11)	0.0340 (10)	0.0682 (13)	−0.0196 (9)	0.0168 (9)	−0.0172 (9)
N5	0.0283 (8)	0.0344 (9)	0.0365 (8)	−0.0138 (7)	0.0058 (6)	−0.0111 (7)
C1	0.0334 (10)	0.0335 (11)	0.0346 (10)	−0.0140 (9)	0.0008 (8)	−0.0060 (8)
C2	0.0354 (10)	0.0323 (10)	0.0335 (10)	−0.0135 (8)	0.0018 (8)	−0.0086 (8)
C3	0.0337 (10)	0.0342 (10)	0.0408 (11)	−0.0117 (8)	0.0014 (8)	−0.0138 (8)
C4	0.0487 (13)	0.0593 (14)	0.0474 (13)	−0.0177 (11)	−0.0071 (10)	−0.0173 (11)
C5	0.0450 (12)	0.0382 (12)	0.0661 (15)	−0.0051 (10)	−0.0028 (11)	−0.0183 (10)
C6	0.0342 (11)	0.0594 (13)	0.0372 (11)	−0.0190 (10)	−0.0018 (8)	−0.0124 (9)
C7	0.0345 (11)	0.0544 (13)	0.0433 (11)	−0.0211 (9)	−0.0007 (9)	−0.0130 (10)
C8	0.0373 (11)	0.0454 (12)	0.0425 (11)	−0.0194 (9)	0.0047 (9)	−0.0068 (9)
C9	0.0326 (10)	0.0356 (11)	0.0358 (10)	−0.0116 (8)	−0.0022 (8)	−0.0065 (8)
C10	0.0379 (11)	0.0301 (10)	0.0379 (11)	−0.0124 (8)	−0.0048 (8)	−0.0061 (8)
C11	0.0458 (12)	0.0398 (12)	0.0368 (11)	−0.0210 (9)	0.0022 (9)	−0.0039 (9)
C12	0.0503 (12)	0.0394 (12)	0.0356 (11)	−0.0151 (10)	−0.0002 (9)	−0.0091 (9)
C13	0.0526 (12)	0.0325 (11)	0.0431 (12)	−0.0162 (9)	−0.0068 (10)	−0.0073 (9)
C14	0.0428 (11)	0.0379 (11)	0.0425 (11)	−0.0202 (9)	−0.0007 (9)	−0.0030 (9)
C15	0.152 (4)	0.064 (2)	0.120 (3)	−0.035 (2)	−0.050 (3)	−0.0171 (19)

### Geometric parameters (Å, º)

**Table:**

Cl1—C10	1.7290 (19)	C4—H4D	0.9600
Cl2—C12	1.734 (2)	C4—H4E	0.9600
Cl3—C13	1.730 (2)	C5—H5A	0.9600
O1—N5	1.4177 (19)	C5—H5B	0.9600
O1—C6	1.455 (2)	C5—H5C	0.9600
O2—C9	1.361 (2)	C6—C7	1.509 (3)
O2—C8	1.436 (2)	C6—H6A	0.9700
O3—C15	1.389 (4)	C6—H6B	0.9700
O3—H3	0.8200	C7—C8	1.506 (3)
N1—C1	1.325 (3)	C7—H7A	0.9700
N1—C3	1.462 (2)	C7—H7B	0.9700
N1—H1	0.866 (10)	C8—H8A	0.9700
N2—C1	1.324 (2)	C8—H8B	0.9700
N2—H2A	0.868 (10)	C9—C14	1.382 (3)
N2—H2B	0.874 (10)	C9—C10	1.396 (3)
N3—C2	1.332 (2)	C10—C11	1.377 (3)
N3—C1	1.349 (2)	C11—C12	1.378 (3)
N4—C2	1.317 (3)	C11—H11	0.9300
N4—H4A	0.883 (10)	C12—C13	1.381 (3)
N4—H4B	0.873 (10)	C13—C14	1.383 (3)
N5—C2	1.369 (2)	C14—H14	0.9300
N5—C3	1.481 (2)	C15—H15A	0.9600
C3—C5	1.514 (3)	C15—H15B	0.9600
C3—C4	1.519 (3)	C15—H15C	0.9600
C4—H4C	0.9600		
			
N5—O1—C6	110.75 (13)	O1—C6—H6A	110.8
C9—O2—C8	118.27 (15)	C7—C6—H6A	110.8
C15—O3—H3	109.5	O1—C6—H6B	110.8
C1—N1—C3	122.30 (16)	C7—C6—H6B	110.8
C1—N1—H1	117.3 (16)	H6A—C6—H6B	108.9
C3—N1—H1	116.2 (16)	C8—C7—C6	112.46 (17)
C1—N2—H2A	121.4 (15)	C8—C7—H7A	109.1
C1—N2—H2B	118.5 (16)	C6—C7—H7A	109.1
H2A—N2—H2B	120 (2)	C8—C7—H7B	109.1
C2—N3—C1	116.03 (16)	C6—C7—H7B	109.1
C2—N4—H4A	119.9 (16)	H7A—C7—H7B	107.8
C2—N4—H4B	114.9 (18)	O2—C8—C7	105.97 (16)
H4A—N4—H4B	124 (2)	O2—C8—H8A	110.5
C2—N5—O1	112.83 (14)	C7—C8—H8A	110.5
C2—N5—C3	117.77 (15)	O2—C8—H8B	110.5
O1—N5—C3	110.56 (13)	C7—C8—H8B	110.5
N2—C1—N1	119.24 (17)	H8A—C8—H8B	108.7
N2—C1—N3	117.74 (17)	O2—C9—C14	125.37 (17)
N1—C1—N3	123.01 (16)	O2—C9—C10	115.84 (17)
N4—C2—N3	120.33 (17)	C14—C9—C10	118.79 (18)
N4—C2—N5	118.00 (17)	C11—C10—C9	120.85 (18)
N3—C2—N5	121.51 (16)	C11—C10—Cl1	119.59 (14)
N1—C3—N5	103.00 (14)	C9—C10—Cl1	119.56 (15)
N1—C3—C5	108.81 (16)	C10—C11—C12	120.00 (18)
N5—C3—C5	109.49 (16)	C10—C11—H11	120.0
N1—C3—C4	111.45 (16)	C12—C11—H11	120.0
N5—C3—C4	111.35 (16)	C11—C12—C13	119.49 (18)
C5—C3—C4	112.31 (18)	C11—C12—Cl2	118.64 (15)
C3—C4—H4C	109.5	C13—C12—Cl2	121.85 (16)
C3—C4—H4D	109.5	C12—C13—C14	120.82 (18)
H4C—C4—H4D	109.5	C12—C13—Cl3	120.49 (16)
C3—C4—H4E	109.5	C14—C13—Cl3	118.68 (15)
H4C—C4—H4E	109.5	C9—C14—C13	120.05 (18)
H4D—C4—H4E	109.5	C9—C14—H14	120.0
C3—C5—H5A	109.5	C13—C14—H14	120.0
C3—C5—H5B	109.5	O3—C15—H15A	109.5
H5A—C5—H5B	109.5	O3—C15—H15B	109.5
C3—C5—H5C	109.5	H15A—C15—H15B	109.5
H5A—C5—H5C	109.5	O3—C15—H15C	109.5
H5B—C5—H5C	109.5	H15A—C15—H15C	109.5
O1—C6—C7	104.82 (15)	H15B—C15—H15C	109.5
			
C6—O1—N5—C2	−107.86 (18)	O1—C6—C7—C8	72.4 (2)
C6—O1—N5—C3	117.90 (17)	C9—O2—C8—C7	−174.20 (16)
C3—N1—C1—N2	−168.24 (18)	C6—C7—C8—O2	−174.17 (16)
C3—N1—C1—N3	12.8 (3)	C8—O2—C9—C14	−3.3 (3)
C2—N3—C1—N2	−173.42 (18)	C8—O2—C9—C10	176.05 (17)
C2—N3—C1—N1	5.6 (3)	O2—C9—C10—C11	−179.53 (18)
C1—N3—C2—N4	−178.78 (19)	C14—C9—C10—C11	−0.1 (3)
C1—N3—C2—N5	5.9 (3)	O2—C9—C10—Cl1	0.1 (2)
O1—N5—C2—N4	19.0 (2)	C14—C9—C10—Cl1	179.54 (15)
C3—N5—C2—N4	149.69 (18)	C9—C10—C11—C12	−0.3 (3)
O1—N5—C2—N3	−165.59 (16)	Cl1—C10—C11—C12	−179.98 (16)
C3—N5—C2—N3	−34.9 (3)	C10—C11—C12—C13	0.5 (3)
C1—N1—C3—N5	−35.7 (2)	C10—C11—C12—Cl2	179.18 (16)
C1—N1—C3—C5	−151.85 (19)	C11—C12—C13—C14	−0.1 (3)
C1—N1—C3—C4	83.8 (2)	Cl2—C12—C13—C14	−178.82 (17)
C2—N5—C3—N1	45.5 (2)	C11—C12—C13—Cl3	179.94 (17)
O1—N5—C3—N1	177.24 (14)	Cl2—C12—C13—Cl3	1.3 (3)
C2—N5—C3—C5	161.15 (17)	O2—C9—C14—C13	179.80 (18)
O1—N5—C3—C5	−67.12 (19)	C10—C9—C14—C13	0.4 (3)
C2—N5—C3—C4	−74.1 (2)	C12—C13—C14—C9	−0.3 (3)
O1—N5—C3—C4	57.68 (19)	Cl3—C13—C14—C9	179.62 (16)
N5—O1—C6—C7	177.50 (15)		

### Hydrogen-bond geometry (Å, º)

**Table:**

*D*—H···*A*	*D*—H	H···*A*	*D*···*A*	*D*—H···*A*
N1—H1···Cl4^i^	0.87 (1)	2.27 (1)	3.1236 (17)	167 (2)
N2—H2*A*···Cl4^ii^	0.87 (1)	2.64 (2)	3.3285 (17)	137 (2)
N2—H2*B*···N3^iii^	0.87 (1)	2.26 (1)	3.122 (2)	170 (2)
N4—H4*A*···Cl4^iv^	0.88 (1)	2.31 (1)	3.1419 (19)	158 (2)
O3—H3···Cl4	0.82	2.35	3.166 (2)	176
C14—H14···O3^v^	0.93	2.53	3.423 (3)	161

Symmetry codes: (i) −*x*+1, −*y*+1, −*z*+1; (ii) *x*+1, *y*, *z*; (iii) −*x*+2, −*y*, −*z*+1; (iv) −*x*+1, −*y*, −*z*+1; (v) −*x*, −*y*+1, −*z*+1.
