# Supplementary figures and images for: Crystal structure of 4,6-di­amino-2,2-dimethyl-3-[3-(2,4,5-tri­chloro­phen­oxy)prop­oxy]-2,3-di­hydro-1,3,5-triazin-1-ium chloride methanol monosolvate
Source: Acta Crystallogr E Crystallogr Commun. 2015 Jul 29;71(Pt 8):o608–9. doi: 10.1107/S205698901501378X (PMC4571422; doi:10.1107/S205698901501378X)

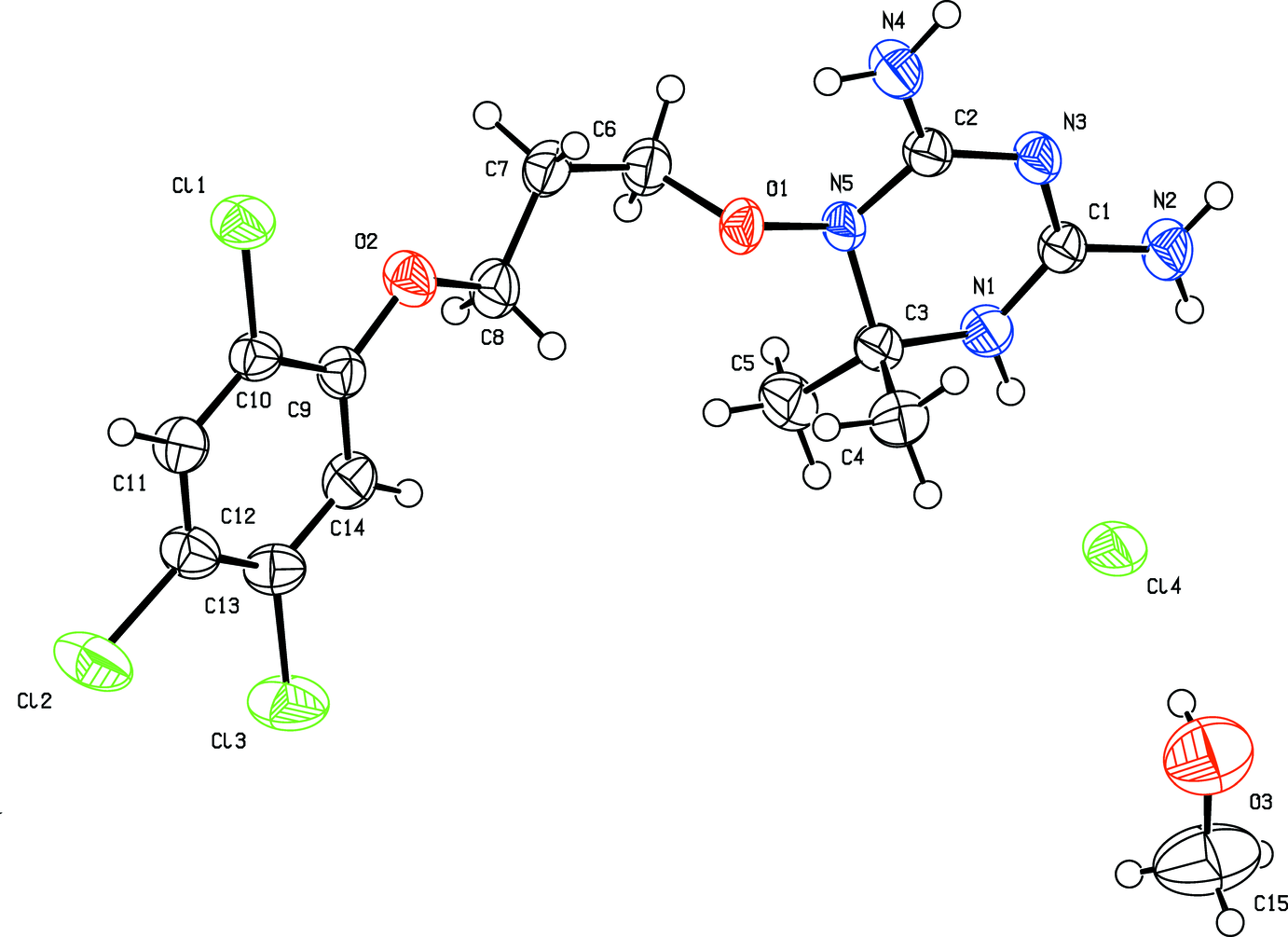

Supplement: Supplementary file 4 [file e-71-0o608-fig1.tif]

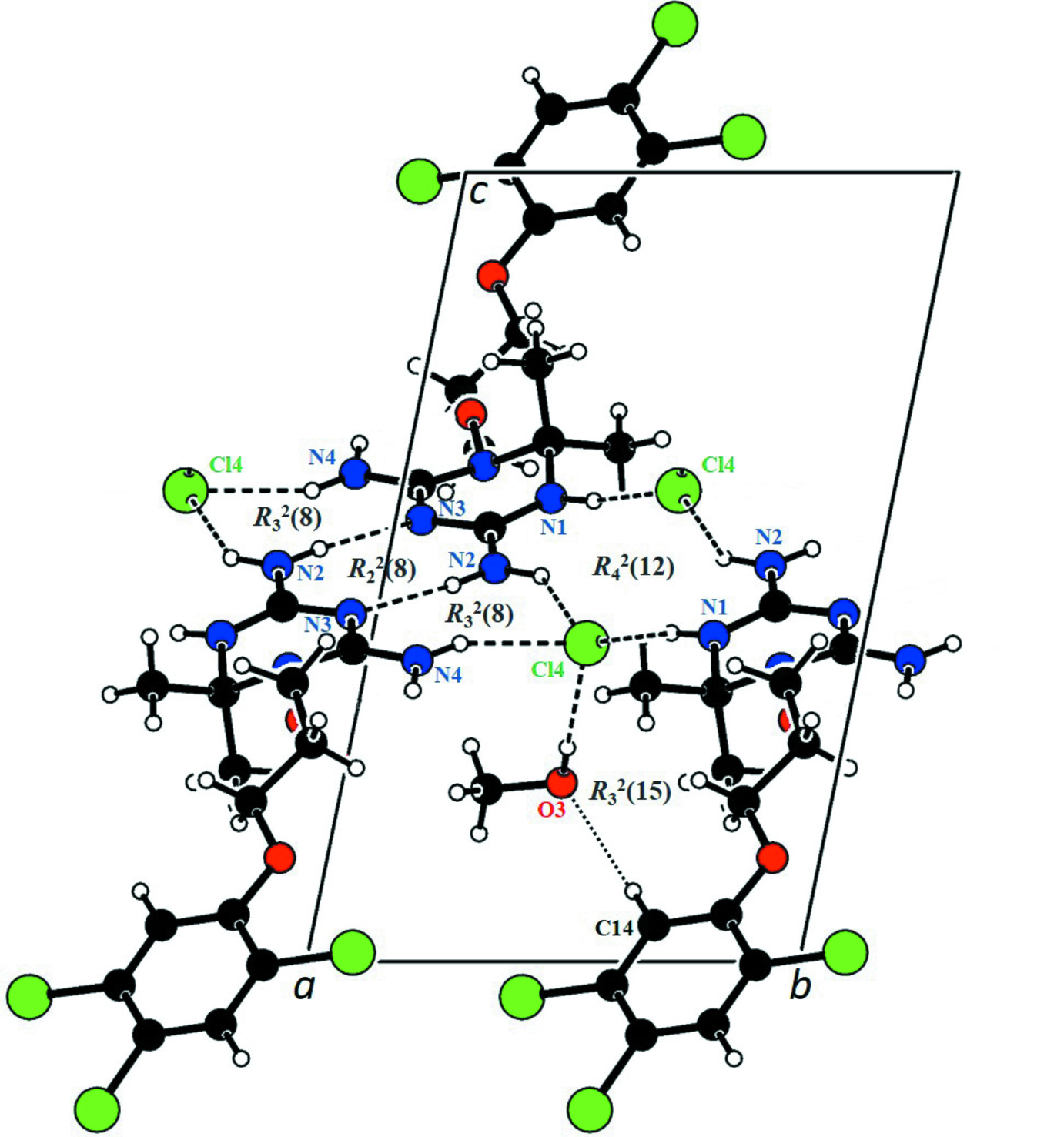

Supplement: Supplementary file 5 [file e-71-0o608-fig2.tif]
